# Incidentalomas are associated with an increase in liver transplantation in patients with cirrhosis: a single-center retrospective study

**DOI:** 10.1186/s12876-022-02379-7

**Published:** 2022-07-10

**Authors:** Pedro Cortés, Hassan M. Ghoz, Fernando Stancampiano, Mohamed Omer, Balkishan Malviya, Andrew W. Bowman, William C. Palmer

**Affiliations:** 1 Division of Community Internal Medicine, Mayo Clinic Florida, Jacksonville, FL 32224 USA; 2 Division of Gastroenterology and Hepatology, Mayo Clinic Florida, 4500 San Pablo Road, Jacksonville, FL 32224 USA; 3 Division of Hospital and Emergency Radiology, Mayo Clinic Florida, Jacksonville, FL 32224 USA

**Keywords:** Liver transplantation, Liver cirrhosis, MRI screening, Incidentaloma, Management

## Abstract

**Background:**

Incidentalomas, defined as incidental findings on imaging, are a growing concern. Our aim was to determine the impact and outcomes of extrahepatic incidentalomas on liver transplantation.

**Methods:**

Patients at a large liver transplant center, who had an initial MRI for hepatocellular carcinoma screening between January 2004 and March 2020 were identified. Clinical data were collected retrospectively. Survival analysis, utilizing Kaplan Meier estimates and Cox proportional hazards regression analysis, was utilized to determine factors associated with liver transplantation.

**Results:**

720 patients were included. NASH (24.9%), HCV (22.1%) and alcohol (20.6%) were the most common causes of cirrhosis. 79.7% of patients had an extrahepatic incidentaloma. Older age and having received a liver transplant by the end of the study were associated with an incidentaloma. MELD was not associated with the presence of an incidentaloma. On univariate Cox proportional hazards regression, male sex, history of moderate alcohol use, smoking history, MELD, and incidentalomas were predictors of liver transplantation. On multivariate analysis, only MELD and the presence of an incidentaloma were found to be significant. Discovery of an incidentaloma was associated with a 30% increase in the risk of liver transplantation. Median time to transplantation did not differ based on the presence on an incidentaloma. Patients with cirrhosis from alcohol or HCV had a significantly shorter median time to transplantation than those with NASH. Renal and pancreatic lesions comprised 91% of all incidentalomas.

**Conclusions:**

In this single-center retrospective study, extrahepatic incidentalomas were common in patients with cirrhosis. The finding of an incidentaloma was associated with a higher risk of liver transplantation despite a similar median time to transplantation if no incidentaloma was discovered.

## Background

The widespread use of abdominal imaging has led to an increased detection of incidental findings, termed incidentalomas, which are defined as incidental radiographic findings that were unexpected or unrelated to the study’s initial purpose [1, 2]. The frequency of incidentalomas varies depending on the imaging study, with the highest detection rates occurring in computed tomography (CT) of the chest (45%), CT enterography (38%), and magnetic resonance imaging (MRI) of the heart (34%) [3]. The most common types of incidentalomas are pituitary [1 in 10], thyroid (up to 50%), pulmonary (8 to 51%), hepatic (15%), pancreatic (2%), adrenal (3 to 4%) and renal (up to 33% in older adults) [4].

Patients with cirrhosis undergo screening for hepatocellular carcinoma (HCC) with transabdominal ultrasound (TUS) as the preferred imaging modality. [5] Obesity, abnormal liver texture, steatosis, technologist’s experience and technical restrictions limit the use of TUS for detecting HCC [6]. The sensitivity for detecting HCC with TUS is 63% for early lesions [7]. In contrast, MRI has a sensitivity of 84.8% for lesions smaller than 2 cm as opposed to 27.3% for TUS [8]. Although, MRI has higher sensitivity, current guidelines by the American Association for the Study of Liver Diseases (AASLD) recommend TUS for HCC surveillance [5, 8]. However, given the limitations of TUS, a growing number of liver transplant centers use MRI as the preferred method for screening [9–11].

Cirrhosis leads to the development of extrahepatic manifestations, including benign and malignant conditions [12, 13]. Benign extrahepatic abnormalities include splenomegaly, ascites, portal hypertension, varices, and bowel and gallbladder edema [13]. The frequency of malignant conditions is increased in patients with cirrhosis compared to the general population and include colorectal and lung cancers (fourfold increase), pancreatic cancer (fivefold), esophageal cancer (eightfold), cholangiocarcinoma (13-fold), and HCC (26-fold) [12]. Given the increased risk of developing extrahepatic abnormalities, the presence of cirrhosis may lead to a higher frequency of benign and malignant incidentalomas.

The significance of incidentalomas in patients with cirrhosis is unclear. With the increasing use of MRI for the screening of HCC, the frequency of incidentalomas may be expected to increase as well. Therefore, we aimed to determine the frequency and outcomes of extrahepatic incidetalomas in patients with cirrhosis undergoing MRI for HCC screening. We also aimed to determine factors that may be associated with extrahepatic incidentalomas, and the impact of incidentalomas on liver transplantation.

## Methods

### Study design and patient selection

The study was approved by the Institutional Review Board. Individual consent for the study was waived. This retrospective study was performed at a large tertiary referral center for liver transplantation and included patients with cirrhosis who underwent MRI as the initial study for HCC screening between January 2004 and March 2020. Patients were excluded if they were younger than 18 years, lacked evidence of cirrhosis, or underwent an initial screening study other than MRI. All information was collected retrospectively, stored in a secure database, and deidentified.

### Data collection

Demographic data included age at time of initial MRI, sex, race, ethnicity, body mass index (BMI), smoking history, moderate alcohol use, prior history of transjugular intrahepatic portosystemic shunt (TIPS), etiology of liver cirrhosis, and liver transplantation status at the end of the study period. Moderate alcohol use was defined per the National Institute on Alcohol Abuse and Alcoholism (NIAAA) criteria of > 14 drinks per week for a male and > 7 drinks per week for a female [14]. Radiographic variables included whether an extrahepatic incidentaloma was discovered, the type of incidental lesion, its size, and whether it was solid or cystic. The radiographic data were obtained through retrospective review of the imaging reports of the MRIs. An extrahepatic incidentaloma was defined as any radiographic finding (such as renal cysts, pancreatic cysts, adrenal adenomas, solid lesions, etc.) that was unexpected or unrelated to the study’s initial purpose. Laboratory data included sodium, total bilirubin, creatinine, international normalized ratio (INR), and model for end-stage liver disease (MELD) at the time of the initial MRI. Pathology data were obtained for patients who underwent biopsy or resection of the incidentaloma. Clinical data regarding the incidentalomas were collected, and included the management approach (observation, surgery), and whether further consultation was pursued.

### Data and statistical analysis

Descriptive statistics for continuous variables were reported as means, and standard deviations. Categorical variables were summarized with number and percentage of patients. Comparisons between patients with and without incidentalomas, and if liver transplantation occurred by the end of the study period, were performed using Student’s t-test, Pearson’s chi-squared test, or Fisher’s exact test as indicated in the accompanying tables.

Survival analysis methods were employed with the time of the initial MRI being defined as time zero. The primary event was defined as the liver transplantation, and time to liver transplantation was recorded for all patients who underwent transplantation during the study period. The secondary event was defined as the discovery of an extrahepatic incidentaloma, and time to incidentaloma discovery was recorded for all patients who had an extrahepatic incidentaloma discovered. Patients were censored at either (1) time of liver transplantation, (2) time at last follow up appointment during the study period, or (3) time of death prior to liver transplantation.

Kaplan Meier curves were constructed for the primary event and comparisons were performed based on (1) the three most common causes of cirrhosis in our cohort, and (2) whether an extrahepatic incidentaloma was discovered. Median time to liver transplantation with 95% confidence intervals (95% CI) were reported. Univariate Cox proportional hazards regression analysis was performed to determine predictors of the primary event of liver transplantation. Variables that were significant at an alpha level of 0.05 were inputted into a multivariate Cox proportional hazards model to adjust for potential confounders. The discovery of an incidentaloma was treated as a time dependent variable and adjusted accordingly based on the time to incidentaloma discovery.

All patients in this cohort had complete data for analysis. All tests were two-sided with an alpha level set at 0.05 for statistical significance. The statistical analysis was performed utilizing BlueSky Statistics software v. 7.10 (BlueSky Statistics LLC, Chicago, IL, USA).

## Results

### Patients characteristics

A total of 720 patients were included in this study. Baseline characteristics of all patients are summarized in Table 1. The three main etiologies of cirrhosis were non-alcoholic steatohepatitis (NASH) (24.9%), hepatitis C (22.1%), and alcohol (20.6%), which together represented 67.6% of all patients. The cohort had 450 males (62.5%), and most patients were White (88.3%). The mean age was 57.7 years (standard deviation [SD] 12.1), mean MELD 14.4 (SD 6.9), and mean BMI 29.4 (SD 6.3). By the end of the study period, a total of 532 patients (73.9%) had undergone liver transplantation, and 574 patients (79.7%) had an extrahepatic incidentaloma discovered.

**Table 1:** Baseline Characteristics of All Patients

Variables	N = 720
Age, year (mean, SD)	57.7 (12.1)
Male Sex, %	450 (62.5%)
BMI, kg/m^2^, (mean, SD)	29.4 (6.3)
Obesity, %	285 (39.6%)
White, %	636 (88.3%)
African American, %	32 (4.4%)
Asian, %	17 (2.4%)
Other Race, %	35 (4.9%)
Hispanic/Latino, %	48 (6.7%)
History of Moderate Alcohol Use, %	142 (19.7%)
Ever smoked, %	399 (55.4%)
Current smoker, %	19 (2.6%)
Sodium, mmol/L (mean, SD)	138 (4.1)
Total Bilirubin, mg/dL (mean, SD)	2.7 (4.2)
INR, (mean, SD)	1.4 (0.4)
Creatinine, mg/dL (mean, SD)	1.0 (0.5)
MELD, (mean, SD)	14.4 (6.9)
MELD ≥ 18, %	201 (27.9%)
MELD ≥ 26, %	49 (6.8%)
*Etiology of Cirrhosis*	
A1AT Deficiency, %	17 (2.4%)
Alcohol, %	148 (20.6%)
Autoimmune, %	45 (6.3%)
Cryptogenic, %	49 (6.81%)
Hemochromatosis, %	9 (1.3%)
Hepatitis B, %	18 (2.5%)
Hepatitis C, %	159 (22.1%)
NASH, %	179 (24.9%)
Other, %	16 (2.2%)
Primary Biliary Cirrhosis, %	32 (4.4%)
Primary Sclerosing Cholangitis, %	45 (6.3%)
Wilson’s Disease, %	3 (0.4%)
History of prior TIPS	38 (5.3%)
Incidentaloma Discovered	574 (79.7%)
Transplanted at End of Study	532 (73.9%)

*SD* standard deviation, *BMI* body mass index, *INR* international standardized ratio, *MELD* model for end-stage liver disease, *A1AT* alpha-1 antitrypsin, *NASH* non-alcoholic steatohepatitis *TIPS* transjugular intrahepatic portosystemic shunt

Comparisons of baseline characteristics are summarized in Table 2 according to whether an extrahepatic incidentaloma was discovered, and in Table 3 according to whether liver transplantation was performed by the end of the study period. Older age, lower total bilirubin, and having been transplanted by the end of the study period were associated with the discovery of an incidentaloma, (*p* < 0.05). Notably, neither the MELD score nor the etiology of cirrhosis was associated with the discovery of an incidentaloma. Multiple variables were associated with liver transplantation, including being a male, having ever smoked, lower serum sodium, higher total bilirubin, creatinine, INR, and MELD, having alcohol or hepatitis C as the etiology of liver cirrhosis, and having had an extrahepatic incidentaloma discovered during the study period.

**Table 2:** Baseline characteristics according to if incidentaloma was discovered on MRI surveillance for HCC

	Incidentaloma Absent N = 146	Incidentaloma Discovered N = 574	*p* value
Age, year (mean, SD)	50.5 (15.5)	59.6 (10.2)	** < 0.001**^**1**^
Male Sex, %	85 (58.2%)	365 (63.6%)	0.231^2^
BMI, kg/m^2^, (mean, SD)	28.6 (6.6)	29.6 (6.2)	0.0848^1^
Obesity, %	56 (38.4%	229 (39.9%)	0.734^2^
White, %	126 (86.3%)	510 (88.9%)	0.392^2^
African American, %	7 (4.8%)	25 (4.4%)	0.822^3^
Asian, %	4 (2.7%)	13 (2.3%)	0.760^3^
Other Race, %	9 (6.2%)	26 (4.5%)	0.393^3^
Hispanic/Latino, %	10 (6.8%)	38 (6.6%)	0.855^3^
History of Moderate Alcohol Use, %	25 (17.1%)	117 (20.4%)	0.377^2^
Ever smoked, %	73 (50.0%)	326 (56.8%)	0.140^2^
Current smoker, %	6 (4.1%)	13 (2.3%)	0.244^3^
Sodium, mmol/L (mean, SD)	137.5 (3.9)	138.1 (4.1)	0.1403^1^
Total Bilirubin, mg/dL (mean, SD)	3.5 (5.4)	2.5 (3.8)	**0.0447**^**1**^
INR, (mean, SD)	1.4 (0.5)	1.4 (0.4)	0.5312^1^
Creatinine, mg/dL (mean, SD)	1.0 (0.6)	1.0 (0.5)	0.6700^1^
MELD, (mean, SD)	15.1 (7.2)	14.2 (6.9)	0.1764^1^
MELD ≥ 18, %	47 (32.2%)	154 (26.8%)	0.215^2^
MELD ≥ 26, %	12 (8.2%)	37 (6.4%)	0.462^3^
Etiology of Cirrhosis			0.931^2^
NASH, %	31 (21.2%)	148 (25.8%)	0.256^2^
Hepatitis C, %	29 (19.9%)	130 (22.6%)	0.469^2^
Alcohol, %	28 (19.2%)	120 (20.9%)	0.645^2^
History of prior TIPS	7 (4.8%)	31 (5.4%)	1.000^3^
Transplanted at end of study	98 (67.1%)	434 (75.6%)	**0.037**^**2**^

**P** values significant at *p* < 0.05 are bolded in the accompanying tables

*MRI* magnetic resonance imaging, *HCC* hepatocellular carcinoma, *SD* standard deviation, *BMI* body mass index, *INR* international standardized ratio, *MELD* model for end-stage liver disease, *NASH* non-alcoholic steatohepatitis, *TIPS* transjugular intrahepatic portosystemic shunt

^1^Student T test, independent samples, two-sided, equal variance not assumed

^2^Pearson’s Chi Square Test

^3^Fisher’s Exact Test

**Table 3:** Baseline characteristics according to if liver transplantation was performed by the end of study period

	Not Transplanted N = 188	Transplanted N = 532	*p* value
Age, year (mean, SD)	59.2 (14.5)	57.2 (11.0)	0.0790^1^
Male Sex, %	102 (54.3%)	348 (65.4%)	**0.007**^**2**^
BMI, kg/m^2^, (mean, SD)	29.3 (6.9)	29.4 (6.0)	0.7777^1^
Obesity, %	74 (39.4%)	211 (39.7%)	0.942^2^
White, %	166 (88.3%)	470 (88.3%)	0.986^2^
African American, %	10 (5.3%)	22 (44.1%)	0.537^3^
Asian, %	5 (2.7%)	12 (2.3%)	0.781^3^
Other Race, %	7 (3.7%)	28 (5.3%)	0.554^3^
Hispanic/Latino, %	8 (4.3%)	40 (7.5%)	0.172^3^
History of Moderate Alcohol Use, %	29 (15.4%)	113 (21.2%)	0.085
Ever smoked, %	90 (47.9%)	309 (58.1%)	**0.015**^**2**^
Current smoker, %	19 (10.1%)	0 (0.0%)	** < 0.001**^**3**^
Sodium, mmol/L (mean, SD)	139.2 (3.0)	137.5 (4.3)	** < 0.001**^**1**^
Total Bilirubin, mg/dL (mean, SD)	1.3 (2.4)	3.2 (4.5)	** < 0.001**^**1**^
INR, (mean, SD)	1.2 (0.3)	1.4 (0.5)	** < 0.001**^**1**^
Creatinine, mg/dL (mean, SD)	1.0 (0.4)	1.0 (0.6)	0.1834^1^
MELD, (mean, SD)	10.4 (4.3)	15.8 (7.1)	** < 0.001**^**1**^
MELD ≥ 18	11 (5.9%)	190 (35.7%)	** < 0.001**^**3**^
MELD ≥ 26	1 (0.5%)	48 (9.0%)	** < 0.001**^**3**^
Etiology of Cirrhosis			0.056^2^
NASH, %	52 (27.7%)	127 (23.9%)	0.302^2^
Hepatitis C, %	31 (16.5%)	128 (24.1%)	**0.031**^**2**^
Alcohol, %	29 (15.4%)	119 (22.4%)	**0.041**^**2**^
History of prior TIPS	6 (3.2%)	32 (6.0%)	0.183^3^
Incidentaloma Discovered	140 (74.5%)	434 (81.6%)	**0.037**

**P** values significant at *p* < 0.05 are bolded in the accompanying tables

*MRI* magnetic resonance imaging, *SD* standard deviation, *BMI* body mass index, *INR* international standardized ratio, *MELD* model for end-stage liver disease, *NASH* non-alcoholic steatohepatitis, *TIPS* transjugular intrahepatic portosystemic shunt

^1^Student T test, independent samples, two-sided, equal variance not assumed

^2^Pearson’s Chi Square Test

^3^Fisher’s Exact Test

### Kaplan Meier analysis

**Fig. 1:**
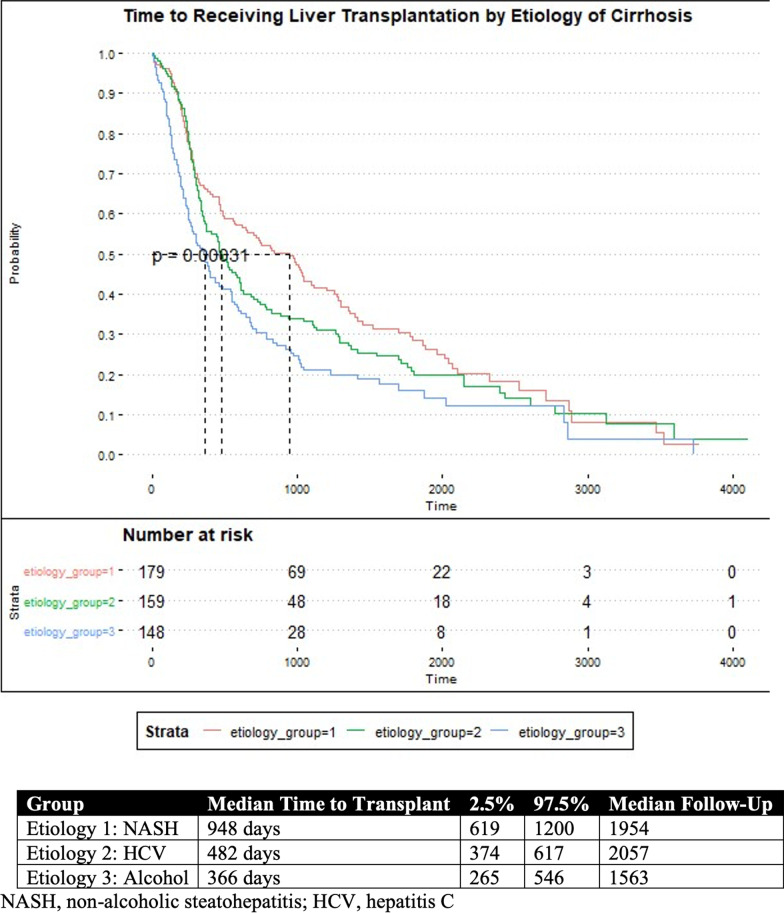
Kaplan–Meier Estimates for being transplanted according to etiology of cirrhosis

**Fig. 2:**
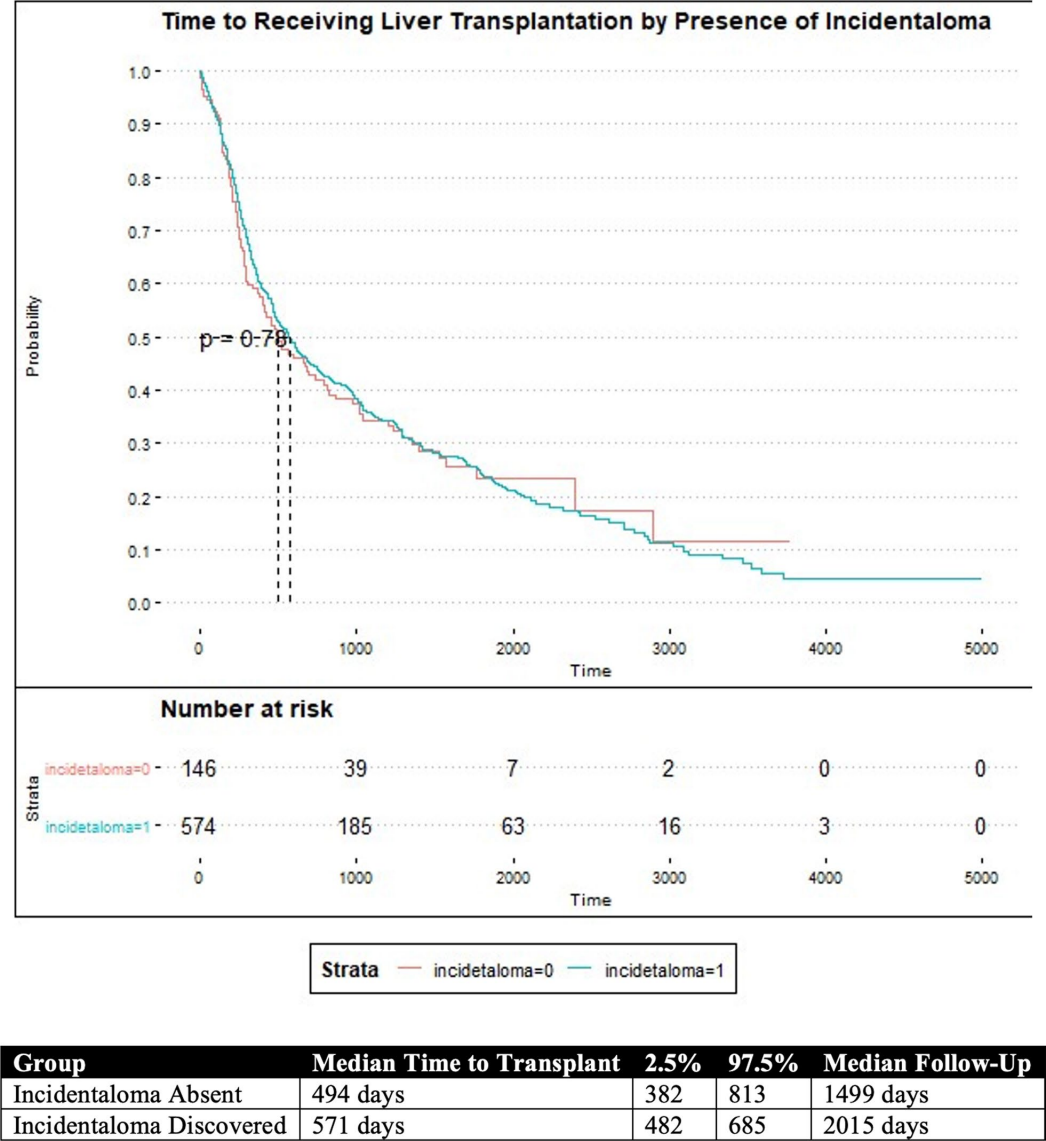
Kaplan–Meier estimates for being transplanted according to incidentaloma being discovered

Kaplan Meier Curves are reported in Figs. 1 and 2. The median time to liver transplantation was statistically different amongst the three most common etiologies of liver cirrhosis, *p* = 0.00831. Patients with alcohol or hepatitis C as the cause of their cirrhosis had a significantly shorter time to liver transplantation than those with NASH. The median time to liver transplantation was 366 days (95% CI: 265–546) and 482 days (95% CI: 374–617) for patients with cirrhosis from alcohol, or hepatitis C, respectively. Patients with NASH had the longest time to liver transplantation at 948 days (95% CI: 619–1200). The median time to liver transplantation did not different according to if an extrahepatic incidentaloma was discovered during the study period, *p* = 0.778. The median time to liver transplantation was 494 days (95% CI: 382–813) without an incidentaloma, and 571 days (95% CI: 482–685) with an incidentaloma.

### Cox proportional hazards regression analysis

The univariate and multivariate models to determine predictors of liver transplantation are reported in Tables 4 and 5, respectively. On univariate analysis, multiple variables were found to be significant at *p* < 0.05, including male sex, having a history of moderate alcohol use, having ever smoked, MELD, and having alcohol as the etiology of liver cirrhosis. The discovery of an extrahepatic incidentaloma was also significantly associated with liver transplantation, HR: 1.3126 (95%: 1.0858–1.5869), *p* = 0.0050, on univariate analysis. These variables were inputted into a multivariate model, and were adjusted for age, BMI, White race, Hispanic/Latino ethnicity, prior history of a TIPS, and NASH cirrhosis. On multivariate analysis, only MELD and the discovery of an extrahepatic incidentaloma remained statistically significant. Every point increase in MELD was associated with a 12% increased risk of liver transplantation (95% CI: 1.1060–1.1380), *p* < 0.001. The discovery of an extrahepatic incidentaloma was associated with a 30% increased risk of liver transplantation (95% CI: 1.0679–1.5763), *p* = 0.0088.

**Table 4:** Univariate Cox proportional hazards regression analysis for predicting liver transplantation

	HR	2.5%	97.5%	p-value
Age, per 1 year	1.0000	0.9900	1.0100	0.9172
Age [18,40) years	0.9100	0.6800	1.2200	0.5397
Age [40,50) years	0.9000	0.6900	1.1800	0.4552
Age [50,65) years	1.1500	0.9700	1.3700	0.0993
Age ≥ 65 years	0.9300	0.7700	1.1200	0.9300
Male Sex	1.2800	1.0700	1.5300	**0.0075**
BMI, per 1 kg/m^2^	0.9900	0.9800	1.0100	0.3536
Obesity	0.9500	0.8000	1.1300	0.5819
White	0.8800	0.6700	1.1400	0.3303
African American	0.8557	0.6300	1.4700	0.8557
Asian	0.9364	0.5800	1.8200	0.9364
Other Race	1.3800	0.9400	2.0200	0.0963
Hispanic/Latino	1.3600	0.9900	1.8800	0.0611
History of Moderate Alcohol Use	1.5200	1.2400	1.8800	** < 0.0001**
Ever smoked	1.2100	1.0200	1.4400	**0.0293**
Sodium, per 1 mmol/L	0.9300	0.9100	0.9500	** < 0.0001**
Total Bilirubin, per 1 mg/dL	1.1500	1.1300	1.1700	** < 0.0001**
INR, per 1 point	2.5300	2.1800	2.9400	** < 0.0001**
Creatinine, per 1 mg/dL point	1.3300	1.1500	1.5500	**0.0001**
MELD, per 1 point	1.1200	1.1100	1.1400	** < 0.0001**
MELD ≥ 18	2.8309	2.3151	3.4616	** < 0.0001**
MELD ≥ 26	2.5241	1.8162	3.5080	** < 0.0001**
Etiology of Cirrhosis	1.2900	1.1300	1.4600	** < 0.0001**
NASH	0.8300	0.6800	1.0100	0.0636
Hepatitis C	1.0700	0.8800	1.3100	0.5049
Alcohol	1.5600	1.2700	1.9100	** < 0.0001**
History of prior TIPS	1.1500	0.8000	1.6500	0.4422
*Binary Time-Dependent Variable*				
Incidentaloma Discovered	1.3126	1.0858	1.5869	**0.0050**

**P** values significant at *p* < 0.05 are bolded in the accompanying tables

*BMI* body mass index, *INR* international standardized ratio, *MELD* model for end-stage liver disease, *NASH* non-alcoholic steatohepatitis, *TIPS* transjugular intrahepatic portosystemic shunt

**Table 5:** Multivariate Cox proportional hazards regression analysis for predicting liver transplantation

	HR	2.5%	97.5%	*p* value
Age, per 1 year	1.0060	0.9983	1.0139	0.1277
Male Sex	1.0948	0.9095	1.3178	0.3384
BMI, per 1 kg/m^2^	0.9973	0.9833	1.0166	0.7110
White	0.9540	0.7241	1.2570	0.7380
Hispanic/Latino	1.3141	0.9436	1.8302	0.1060
History of Moderate Alcohol Use	1.0178	0.8052	1.2865	0.8827
Ever smoked	1.1609	0.9656	1.3956	0.1125
MELD, per 1 point	1.1219	1.1060	1.1380	** < 0.001**
NASH	0.9365	0.7464	1.1751	0.5712
History of prior TIPS	0.8839	0.6120	1.2766	0.5106
*Binary Time-Dependent Variable*				
Incidentaloma Discovered	1.2975	1.0679	1.5763	**0.0088**

**P** values significant at *p* < 0.05 are bolded in the accompanying tables

*BMI* body mass index, *MELD* model for end-stage liver disease, *NASH* non-alcoholic steatohepatitis, *TIPS* transjugular intrahepatic portosystemic shunt

### Extrahepatic incidentalomas

A total of 690 extrahepatic lesions were found amongst 720 patients, Table 6. Approximately 80% of patients had an incidentaloma discovered, and 106 patients (18.5%) had more than one extrahepatic incidentaloma discovered. Most of these incidental findings included renal (60.9%) and pancreatic lesions (30.1%), representing 91.0% of all incidentalomas. Most incidentalomas were managed with observation (98.8%). Amongst renal and pancreatic incidentalomas, 1.2% and 52.4% were referred to urology or gastroenterology, respectively, Table 7. Amongst all incidentalomas, only 16 underwent biopsy or resection, of which, nearly 50% were pancreatic lesions. A total of 7 malignant incidentalomas were diagnosed.

**Table 6:** Types of extra-hepatic incidentalomas

Patients with Incidentalomas, N = 574	Total, %
Total number of Incidentalomas	690
Patients with multiple incidentalomas	106 (18.5%)
*Types of incidentalomas*	
Kidney	420 (60.9%)
Pancreas	208 (30.1%)
Spleen	25 (3.6%)
Adrenal	11 (1.6%)
Gallbladder	11 (1.6%)
Pelvic	9 (1.3%)
Bone	4 (0.6%)
Stomach	2 (0.3%)
Number of incidentalomas undergoing biopsy/resection	16 (2.3%)
Pancreas	7 (47.1%)
Kidney	3 (17.6%)
Gallbladder	4 (23.5%)
Pelvis	1 (5.9%)
Stomach	1 (5.9%)
Number of malignancies detected	7 (1.0%)
Gastric Neuroendocrine Tumor	1 (14.3%)
Cholangiocarcinoma	2 (28.6%)
Renal cell carcinoma	3 (42.9%)
Pancreatic adenocarcinoma	1 (14.3%)
*Management of incidentalomas*	
Observation	682 (98.8%)
Surgery, alone	5 (0.7%)
Chemotherapy/Radiation, alone	2 (0.3%)
Surgery and Chemotherapy/Radiation	1 (0.1%)

**Table 7:** Features of Kidney and Pancreatic Incidentalomas

Incidentaloma, N = 690	Total, %
Kidney or Pancreatic	628 (91.0%)
Kidney	420
*Type of Lesion*	
Simple Cyst	417 (99.3%)
Solid Lesion	3 (0.7%)
*Size of Lesion*	
< 1 cm	396 (94.3%)
[1,3) cm	15 (3.6%)
[3, 5) cm	3 (0.7%)
≥ 5 cm	6 (1.4%)
Referred to Urology	5 (1.2%)
Pancreas	208
*Type of Lesion*	
IPMN, Cystic Lesion	207 (99.5%)
Solid Lesion	1 (0.5%)
*Size of Lesion*	
< 1 cm	169 (81.3%)
[1,3) cm	36 (17.3%)
[3, 5) cm	2 (1.0%)
≥ 5 cm	1 (0.5%)
Referred to Gastroenterology	109 (52.4%)
Underwent EUS	12 (5.8%)

*IPMN* intraductal papillary mucinous neoplasm, *EUS* endoscopic ultrasound

The overwhelming majority of renal incidentalomas were simple cysts (99.3%) and characterized as Bosniak 1 or 2 by the reading radiologist. The three solid renal lesions were biopsied and found to be renal cell carcinoma. Similarly, most pancreatic lesions were characterized as intraductal papillary mucinous neoplasms (IPMN) (99.5%) by the reading radiologist and were sub-centimeter (81.3%). Only 12 of all pancreatic lesions underwent endoscopic ultrasound (EUS). Table 8 summarizes the findings of EUS and the subsequent pathology. Only the solid pancreatic lesion was found to be malignant, whereas the other biopsied pancreatic lesions were confirmed to be benign.

**Table 8:** Features of pancreatic lesions that underwent endoscopic ultrasound

MRI Findings	CA 19–9 (U/mL)	EUS Findings	Biopsy	Pathology
Numerous subcentimeter non-enhancing cysts, likely side-branch IPMNs	N/A	Mild-moderate chronic pancreatitis	No	N/A
1.4 × 2.2 cm cystic lesion at pancreatic head, likely IPMN	105	Multicystic, septated, 22 × 15 mm lesion	Yes	Malignancy absent, mucinous epithelium
Multilobulated pancreatic tail cystic lesion, 2.4 × 2.9 cm	N/A	Septated lesion, 28 mm, side-branch IPMN	Yes	Malignancy absent, mucinous epithelium
Parenchymal atrophy, innumerable tiny cysts; irregular main duct	N/A	Many benign cysts in tail; largest 7 mm	No	N/A
3.3 × 2.3 cm septated cystic lesion at pancreatic neck	6	Multiloculated 2.75 × 1.98 cm cyst at neck	Yes	Malignancy absent, mucinous epithelium
Numerous unilocular cysts at head, largest 14 mm	255	12 × 10 mm cyst in pancreatic head	Yes	Malignancy absent, mucinous epithelium
1.7 cm hypoenhancing head mass	103	1.7 cm pancreatic head mass	Yes	Adenocarcinoma
3 mm cystic lesion at uncinate process	N/A	8 × 8 mm uncinate cystic lesion	No	N/A
Few sub-5 cm cystic foci in pancreas	44	Few cysts in the pancreatic head	No	N/A
7–8 mm enhancing lesion at uncinate process, suspicious for NET	35	Multiple cystic lesions at uncinate, 10 × 10 mm	Yes	Malignancy absent, mucinous epithelium
Small cystic lesions, likely side-branch IPMNs	29	Pancreatic head cysts, 3 × 3 mm, no mass	No	N/A
Small cystic lesions, largest is 8 × 12 mm, likely IPMN	N/A	25 × 17 mm pancreatic head cystic lesion	Yes	Malignancy absent, mucinous epithelium

*MRI* magnetic resonance imaging; CA 19–9, carbohydrate antigen 19–9 (reference range: < 55 U/mL); *EUS* endoscopic ultrasound, *N/A* not available, *NET* neuroendocrine tumor, *IPMN* intraductal papillary mucinous neoplasm

A total of 7 cancers were diagnosed (1.0% of all incidentalomas). Table 9 summarizes the features of the malignant incidentalomas. The malignant lesions include three renal cell carcinomas (RCC) (42.9%), two cholangiocarcinomas (28.6%), one pancreatic adenocarcinoma (PDAC), and one gastric neuroendocrine tumor (NET). None of the patients had metastases. Four out of seven patients eventually underwent liver transplantation. One patient underwent neoadjuvant chemotherapy for cholangiocarcinoma before having liver transplantation for curative intent. Only one patient died from a malignant incidentaloma.

**Table 9:** Features of malignant incidentalomas

Incidental Finding	Size	Pathology	Treatment	Transplanted
Gastric solid lesion	< 1 cm	Well-differentiated NET	EMR, Cured	No
Gallbladder solid lesion	1–3 cm	Cholangiocarcinoma	Chemoradiation, Died	No
Renal solid lesion	> 5 cm	Renal cell carcinoma, clear cell	Nephrectomy, Cured	Yes
Gallbladder solid lesion	1–3 cm	Cholangiocarcinoma	Neoadjuvant Chemotherapy, Cured with transplantation	Yes
Renal solid lesion	1–3 cm	Renal cell carcinoma, papillary	Nephrectomy, Cured	Yes
Renal solid lesion	< 1 cm	Renal cell carcinoma, clear cell	Nephrectomy, Cured	Yes
Pancreatic solid lesion	1–3 cm	Pancreatic adenocarcinoma	Chemotherapy and Surgery, Cured	No

*NET* neuroendocrine tumor, *EMR* endoscopic mucosal resection

## Discussion

The main findings of our study were: (1) neither MELD nor the etiology of cirrhosis were associated with extrahepatic incidentalomas, (2) only MELD and the discovery of an incidentaloma were predictors of liver transplantation after adjusting for potential confounders, (3) the discovery of an incidentaloma did not affect the median time to liver transplantation, and (4) the discovery of a malignant incidentaloma was rare but led to cures in all but one patient.

Few studies have studied the frequency and outcomes of incidentalomas discovered on screening MRI. Ibrahim et al*.,* reported the frequency of incidentalomas on MRI was assessed in individuals at high risk (IARs) for PDAC [15]. A total of 459 incidentalomas were discovered, eleven of which were cancerous (1.9%) and six metastatic at diagnosis. The early detection of cancer was beneficial in five of eleven IARs. In another study, whole-body MRI was performed to detect the frequency of incidentalomas in 118 healthy volunteers (mean age 47.4 years, range 20–81) [16]. Seventy percent of volunteers had an incidental finding detected, and a total of 103 benign lesions were found. Only 2 malignant lesions (1.9%) were found. In contrast to others, our study is the first to determine the frequency, and clinical outcomes of extrahepatic incidentalomas in patients with cirrhosis undergoing MRI for the screening of HCC. Like prior studies, we found that incidental malignancies were rare, and that their detection led to cure in most patients.

To our knowledge, our study is the only one to have explored the association between extrahepatic incidentalomas and liver transplantation. It remains unclear why the discovery of an incidentaloma was found to be a significant predictor of liver transplantation. Incidental findings on imaging have been reported to lead to a “cascade effect”, whereby the incidentaloma leads to further testing by providers [17, 18]. In a national survey of U.S. physicians, 99.4% of respondents reported having experienced “cascades of care” whereby incidental findings led respondents to perform additional testing [19]. In a retrospective study of 592 patients with head and neck squamous cell cancer (HNSCC) who underwent staging with PET/CT, incidental findings occurred in 61.5% of patients. The discovery of an incidental finding was a significant predictor of treatment delay in this cohort [20]. Liver transplantation requires an extensive evaluation of the recipient’s comorbidities and contraindications to transplantation, which include extrahepatic malignancy [21]. Although non-significant, patients with incidentalomas appeared to have a longer time to liver transplantation than those without incidentalomas, indicating a possible treatment delay due to more extensive evaluation as seen in the study of patients with HNSCC.

Although current guidelines by the AASLD recommend TUS over MRI for HCC screening, the former has multiple limitations, that may lead to failure to detect early-stage cancer when it is the most curable [22]. Studies suggest MRI has a higher sensitivity for detecting early-stage HCC and may be more cost-effective in certain populations [5, 8–11, 23]. Curing HCC becomes increasingly difficult when the size of the HCC becomes greater than 2–2.5 cm [24–26]. TUS has a sensitivity between 27.3 and 63% for detecting early-stage lesions that are less than 2 cm [7, 8]. Additionally, abnormal liver parenchyma, obesity, ascites or hepatic steatosis may further decrease the sensitivity for the detection of HCC by attenuating ultrasound waves [22]. By 2030, 51% of the United states population will be obese [27]. With the increasing incidence of obesity, NASH and steatosis are likely to increase as well, further limiting the utility of TUS. Therefore, the use of MRI for screening of HCC will likely increase in the future, leading to a higher number of extrahepatic incidentalomas.

Our study has several limitations. First, the retrospective nature of the study limited our ability to determine the thought process behind the management of extrahepatic incidentalomas, which may have led to potential confounders. These potential confounders could have contributed to the “cascade effect” leading to higher rates of liver transplantation in those with incidentalomas. Second, most patients in our cohort were White, therefore, our findings on the benign nature of extrahepatic incidentalomas may not be generalizable to other racial backgrounds, who have a higher incidence of certain cancers, such as lung, prostate and colorectal malignancies in African Americans [28]. Third, we were unable to measure, and adjust for the potential confounding of MRI sensitivity over time. Over the long course of this study, the MRI scanners in our institution have been upgraded and replaced several times, and it is likely that increases in magnet field strength and improved imaging software application that were used in our most recent scans allow for better detection of smaller extrahepatic incidentalomas. Fourth, the average MELD score of our patient population was relatively low and may not be generalizable to other liver transplant programs across the country. Fifth, the presence of incidentalomas was determined through retrospective review of the imaging report, and not by manual re-read of every scan. This approach was felt to be sufficient given imaging reports are the standard means by which imaging findings are communicated in clinical practice. Finally, although our study identified a large proportion of patients with cirrhosis who underwent MRI for HCC surveillance at our institution, it was not exhaustive of all patients.

We believe our study provides insight into the impact of extrahepatic incidentalomas on liver transplantation. Given the worsening obesity epidemic, the prevalence of liver disease and HCC is expected to increase [29]. With higher failure rates for HCC detection in obese patients, TUS may not be the preferred screening modality for HCC in the coming years, leading to increased utilization of MRIs and a higher prevalence of extrahepatic incidentalomas. The increase in incidentalomas may have a “cascade effect” which could potentially lead to increases in the total number of liver transplantation, thereby, increasing the demand of a limited resource.

## Conclusions

In this large retrospective study of patients with cirrhosis at a large liver transplantation program, most patients had an extrahepatic incidentaloma discovered on routine MRI for the screening of HCC. Renal and pancreatic cysts were the most common incidentalomas discovered and most were managed conservatively with observation. One percent of extrahepatic incidentalomas were cancerous. The discovery of an extrahepatic incidentaloma was associated with an increased risk of liver transplantation after adjusting for multiple covariates relevant to the cirrhosis population. Although the exact reason for this association remains unclear, the “cascade effect” may explain this observation. Further studies at other liver transplantation centers are needed to validate this finding. With the rising obesity epidemic, the use of MRI for HCC screening will likely continue to increase leading to an increased incidence in incidentalomas, and possibly, more liver transplantation.

## Data Availability

The de-identified datasets used and/or analyzed during the current study are available from the corresponding author on reasonable request. Data, analytic methods, and study material could be made available upon request to the corresponding author and approval by the Mayo Clinic Institutional Review Board. Legal restrictions apply to these data.

## Abbreviations

MRI: Magnetic resonance imaging
NASH: Non-alcoholic steatohepatitis
HCV: Hepatitis C virus
MELD: Model for end-stage liver disease
CT: Computed tomography
HCC: Hepatocellular carcinoma
TUS: Transabdominal ultrasound
AASLD: American association for the study of liver diseases
BMI: Body mass index
TIPS: Transjugular intrahepatic portosystemic shunt
NIAAA: National Institute on alcohol abuse and alcoholism
INR: International normalized ratio
IPMN: Intraductal papillary mucinous neoplasms
EUS: Endoscopic ultrasound
RCC: Renal cell carcinoma
PDAC: Pancreatic ductal adenocarcinoma
NET: Neuroendocrine tumor
IARs: Individuals at high risk
HNSCC: Head and neck squamous cell cancer

## References

[1] Davenport C, Liew A, Doherty B, Win HH, Misran H, Hanna S (2011). The prevalence of adrenal incidentaloma in routine clinical practice. Endocrine.

[2] Ng VW, Ma RC, So WY, Choi KC, Kong AP, Cockram CS (2010). Evaluation of functional and malignant adrenal incidentalomas. Arch Intern Med.

[3] O'Sullivan JW, Muntinga T, Grigg S, Ioannidis JPA (2018). Prevalence and outcomes of incidental imaging findings: umbrella review. BMJ.

[4] Hitzeman N, Cotton E (2014). Incidentalomas: initial management. Am Fam Physician.

[5] Heimbach JK, Kulik LM, Finn RS, Sirlin CB, Abecassis MM, Roberts LR (2018). AASLD guidelines for the treatment of hepatocellular carcinoma. Hepatology.

[6] Dănilă M, Sporea I (2014). Ultrasound screening for hepatocellular carcinoma in patients with advanced liver fibrosis. An Overview Med Ultrason.

[7] Singal A, Volk ML, Waljee A, Salgia R, Higgins P, Rogers MA (2009). Meta-analysis: surveillance with ultrasound for early-stage hepatocellular carcinoma in patients with cirrhosis. Aliment Pharmacol Ther.

[8] Kim SY, An J, Lim YS, Han S, Lee JY, Byun JH (2017). MRI with liver-specific contrast for surveillance of patients with cirrhosis at high risk of hepatocellular carcinoma. JAMA Oncol.

[9] An JY, Peña MA, Cunha GM, Booker MT, Taouli B, Yokoo T (2020). Abbreviated MRI for hepatocellular carcinoma screening and surveillance. Radiographics.

[10] Ganne-Carrié N, Piscaglia F (2020). Non-enhanced MRI surveillance for HCC: A new tool for all, none or selected patients at risk?. J Hepatol.

[11] Kim HL, An J, Park JA, Park SH, Lim YS, Lee EK (2019). Magnetic resonance imaging is cost-effective for hepatocellular carcinoma surveillance in high-risk patients with cirrhosis. Hepatology.

[12] Kalaitzakis E, Gunnarsdottir SA, Josefsson A, Björnsson E (2011). Increased risk for malignant neoplasms among patients with cirrhosis. Clin Gastroenterol Hepatol.

[13] Sangster GP, Previgliano CH, Nader M, Chwoschtschinsky E, Heldmann MG (2013). MDCT imaging findings of liver cirrhosis: spectrum of hepatic and extrahepatic abdominal complications. HPB Surg.

[14] National Insitute on Alcohol Abuse and Alcoholism (2005). Helping patients who drink too much: a clinician’s guide.

[15] Ibrahim IS, Brückner C, Carrato A, Earl J, Inderson A, de Vos Tot Nederveen Cappel WH (2019). Incidental findings in pancreas screening programs for high-risk individuals: results from three European expert centers. United Eur Gastroenterol J.

[16] Ulus S, Suleyman E, Ozcan UA, Karaarslan E (2016). Whole-body MRI screening in asymptomatic subjects; preliminary experience and long-term follow-up findings. Pol J Radiol.

[17] Mold JW, Stein HF (1986). The cascade effect in the clinical care of patients. N Engl J Med.

[18] Westbrook JI, Braithwaite J, McIntosh JH (1998). The outcomes for patients with incidental lesions: Serendipitous or iatrogenic?. AJR Am J Roentgenol.

[19] Ganguli I, Simpkin AL, Lupo C, Weissman A, Mainor AJ, Orav EJ (2019). Cascades of care after incidental findings in a US National Survey of physicians. JAMA Netw Open.

[20] Schoonbeek RC, Bult FFS, Plaat BEC, Witjes MJH, van der Hoorn A, van Dijk BAC (2021). Incidental findings during the diagnostic work-up in the head and neck cancer pathway: effects on treatment delay and survival. Oral Oncol.

[21] Martin P, DiMartini A, Feng S, Brown R, Fallon M (2014). Evaluation for liver transplantation in adults: 2013 practice guideline by the American Association for the Study of Liver Diseases and the American Society of Transplantation. Hepatology.

[22] Sherman M (2014). Limitations of screening for hepatocellular carcinoma. Hepat Oncol.

[23] Lim J, Singal AG (2019). Surveillance and diagnosis of hepatocellular carcinoma. Clin Liver Dis (Hoboken).

[24] Ikai I, Arii S, Kojiro M, Ichida T, Makuuchi M, Matsuyama Y (2004). Reevaluation of prognostic factors for survival after liver resection in patients with hepatocellular carcinoma in a Japanese nationwide survey. Cancer.

[25] Sala M, Llovet JM, Vilana R, Bianchi L, Solé M, Ayuso C (2004). Initial response to percutaneous ablation predicts survival in patients with hepatocellular carcinoma. Hepatology.

[26] Shah SA, Cleary SP, Wei AC, Yang I, Taylor BR, Hemming AW (2007). Recurrence after liver resection for hepatocellular carcinoma: risk factors, treatment, and outcomes. Surgery.

[27] Finkelstein EA, Khavjou OA, Thompson H, Trogdon JG, Pan L, Sherry B (2012). Obesity and severe obesity forecasts through 2030. Am J Prev Med.

[28] Zavala VA, Bracci PM, Carethers JM, Carvajal-Carmona L, Coggins NB, Cruz-Correa MR (2021). Cancer health disparities in racial/ethnic minorities in the United States. Br J Cancer.

[29] Hagström H, Tynelius P, Rasmussen F (2018). High BMI in late adolescence predicts future severe liver disease and hepatocellular carcinoma: a national, population-based cohort study in 1.2 million men. Gut.

